# Tissue Reactions to Various Suture Materials Used in Oral Surgical Interventions

**DOI:** 10.5402/2012/762095

**Published:** 2012-05-08

**Authors:** Fawad Javed, Mansour Al-Askar, Khalid Almas, Georgios E. Romanos, Khalid Al-Hezaimi

**Affiliations:** ^1^Engineer Abdullah Bugshan Research Chair for Growth Factors and Bone Regeneration, King Saud University, P.O. Box 60169, Riyadh 11545, Saudi Arabia; ^2^Department of Periodontics and Community Dentistry, King Saud University, Riyadh 11545, Saudi Arabia; ^3^Division of Periodontology, University of Connecticut, Farmington, CT 06032, USA; ^4^Division of Periodontology, Eastman Institute for Oral Health, University of Rochester, Rochester, NY 14620, USA

## Abstract

A variety of suture materials are available for primary wound closure following oral surgical procedures. The aim was to review the tissue reactions to the various suture materials used in oral surgical interventions. Databases were searched using the following keywords: cotton, nylon, polyglecaprone 25, polytetrafluoroethylene (ePTFE), Polyglactin 910, polyglycolic acid (PGA), polylactic acid, silk, surgery, suture, and tissue reaction. Articles published only in English language were included. Seventeen studies were included. Two studies reported that polyglecaprone 25 had positive effects on wound-healing as compared to silk. Six studies reported that silk elicits more intense tissue inflammatory response and delayed wound healing as compared to other suture materials (including ePTFE, polyglecaprone-25, PGA, and nylon). Polyglactin 910 sutures were associated with the development of stitch abscess in one clinical study. Eight studies reported that tissue reactions are minimal with nylon sutures. Tissue reactions to suture materials used for oral surgical interventions may vary depending on the surface properties and bacterial adherence properties of the material.

## 1. Introduction

Most oral surgical interventions require primary wound closure using a previously raised flap. For this purpose, a variety of suture materials are available which may be classified upon their origin (organic and synthetic) or according to their durability in host tissues (absorbable and nonabsorbable) [1, 2]. The essential features of suture material include (1) knot safety, (2) stretch capacity, (3) tissue reactivity, and (4) wound safety. Besides the adopted surgical and suturing technique, the choice of suture material may also influence the healing of the incised soft tissues [1–3]. In their study, Vastardis and Yukna [4] reported three case reports of complications after the use of an subepithelial connective tissue graft where an abscess occurred following the initial healing phase. This study [4] concluded that a stitch abscess or reaction to the suture material used for the submerged sutures could be a possible cause of the abscesses. Thus the selection of the suture material should be brought under consideration during treatment planning for oral surgical interventions.

Tissue reaction is reflected through an inflammatory response, which develops during the first two to seven days after suturing the tissue [1–3]. Several studies published over the past four decades have reported that synthetic materials exhibit a superior behavior to oral tissues in terms of tissue inflammatory reactions compared to nonsynthetic suture materials [3–19]. Suture materials that have been frequently investigated in terms of tissue reactions include cotton, braided silk, polyester, nylon, and cat gut; however, the study outcomes remain debatable. Polyester sutures have been reported to cause a mild inflammatory reaction whereas cotton threads have been associated with an intense tissue inflammatory response [15–17]. Other commercially available suture materials include polyglycolic acid (PGA) and polyglactin 910 (derived from copolymerization of glycosides and lactides) and have been labeled as “desirable suture materials” [1, 15, 20]; nevertheless, controversy persists over the efficacy of suture materials. Sortino et al. [8] reported the bacterial count over the braided silk and PGA sutures to be similar; conversely, other studies have reported that silk sutures are more susceptible to bacterial invasion and severe tissue inflammatory reactions compared to other suturing products [14–17]. However, in terms of cost-effectiveness, silk continues to enjoy its status as an “inexpensive” suture material as compared to other nonabsorbable suture materials [2].

Since the choice of the suture material used in oral surgical interventions may play a role in optimal postsurgical wound healing, the present study aimed to review the tissue reactions to the various suture materials used in oral surgical interventions.

## 2. Materials and Methods

### 2.1. Focused Question

The addressed focused question was: do the tissues react to the various suture materials used in oral surgical interventions?

### 2.2. Eligibility Criteria

The following eligibility criteria were imposed: (1) clinical and experimental studies; (2) intervention: suture materials used in oral surgery; (3) reference list of potentially relevant original and review research studies; and (4) articles published only in English language. Unpublished articles, letters to the editor, and historical reviews were excluded.

### 2.3. Search Strategy

The authors explored the MEDLINE-PubMed databases of the National Library of Medicine, Bethesda, Maryland, for articles addressing the focused question. Databases were searched from 1968 up to and including June 2010 using the following keywords in different combinations: cotton, dental, flap, inflammation, materials, nylon, oral, periodontal, polyglecaprone, polytetrafluoroethylene, Polyglactin 910, polyglycolic acid, polylactic acid, silk, surgery, suture and tissue reaction.

The next step was to hand-search the reference lists of original and review studies that were found to be relevant in the first step, and once again, any disagreement between the authors was resolved via discussion. The initial search yielded 66 studies. Scrutiny of the titles and abstracts reduced the number of studies to seventeen [3–5, 7–19] which were processed for data extraction (Table 1). Forty-nine studies that did not fulfill the eligibility criteria (as described previously) were excluded.

## 3. Results

### 3.1. Characteristics of Included Studies

All the 17 studies [3–5, 7–19] included in the present literature review were either carried out at universities or at healthcare centers. Six studies [4, 8–10, 12, 15] were clinical and 11 studies [3, 5, 7, 11, 13, 14, 16, 19] had an experimental research design. The experimental studies were performed on male Wistar rats, Rhesus monkeys and Beagle dogs [3, 5, 7, 11, 13, 14, 16–19]. In all clinical studies [4, 8–10, 12, 15], the participants were systemically healthy, whereas in one experimental study [7], efficacies of various suture materials were investigated in diabetic male Wistar rats. The investigated suture materials were catgut, cotton, nylon, perlon, polyester, polyglecaprone 25, PGA, expanded polytetrafluoroethylene (ePTFE), braided silk, and steel. In eight studies [4, 10, 11, 13–15, 17], involving periodontal surgical interventions, tissue reactions were compared between braided silk and other suture materials including cotton, chromic, nylon, and polyglactin 910. In four studies [3, 5, 18, 19], oral surgical procedures were performed on the buccal mucosae and tongues of beagle dogs and the sutures materials under investigation included silk, cotton, polyester, steel, and chromic.

Two studies [7, 9] reported that polyglecaprone 25 had positive effects on wound healing and exhibited lesser numbers of adherent bacteria as compared to braided silk. Six studies [9–11, 13, 15, 16] (five clinical [9, 10, 13, 15, 16] and one experimental [11]) reported that braided silk elicits more intense tissue inflammatory response and delayed wound healing as compared to other suture materials (including ePTFE, polyglecaprone 25, PGA, and nylon). In a study by Vastardis and Yukna [4], three case reports were presented where the occurrence of stitch abscess was associated with Polyglactin 910 sutures. In their experimental study, Yilmaz et al. [7] reported that silk and chromic gut are well tolerated in diabetic rats whereas Selvig et al. [14] reported bacterial invasion to be common in these materials, particularly in braided silk sutures. Four studies [3, 16, 17] associated cotton sutures with intense tissue reactions. Eight studies [3, 5, 15–19] reported that nylon sutures provide the best biological results. These studies [15–17], also reported the least inflammatory response. Castelli et al. [17] compared the tissue inflammatory responses induced by silk, cotton and nylon, and the results showed that nylon sutures did not elicit any form of inflammatory response in oral tissues compared to silk and cotton.

## 4. Discussion

Several suture materials are available for dental and medical surgical procedures; however, it is essential for surgeons to be aware of the nature of the suture material, the biologic processes of healing, and the interaction of the suture material with the surrounding tissues. This is a critical issue because the surgeon must ensure that a suture will retain its strength until the tissues of the previously raised surgical flaps recover sufficient strength to keep the wound edges together. To date, research data regarding the efficacies of various materials remains debatable and inconsistent. Thus the present study attempted to review the tissue reactions to different suture materials used in oral surgical interventions.

Traditionally, silk has been the mostly used suture material for dental and several other surgical procedures [21]. Even though silk is inexpensive and easy to handle as compared to other nonabsorbable suture materials [19, 22]; the authors believe that it should not be considered as a “material of choice” for oral surgical interventions. Studies on oral tissue reactions to sutures have revealed constant inflammatory reactions, which are most prominent with silk and cotton and minimal with others including nylon, polyester, ePTFE, polyglecaprone 25 and PGA [3, 5, 7–19]. A histological study [15] compared the oral tissue reactions to various suture materials. The results showed the presence of a large number of neutrophilic polymorphonuclear leukocytes in the premises of silk sutures which were less intense in oral tissues farther from silk sutures [15]. Another finding was that fibroblasts and new capillaries formed at a slower pace in the oral tissues in the vicinity of silk sutures compared to tissues farther from the silk sutures. This may be a justification for the delayed healing and severe tissue reactions associated with silk sutures.

Another factor that may instigate tissue reactions is the capability of bacteria to adhere to various suture materials. In their *in vitro *study, Katz et al. [23] investigated the capability of bacteria to adhere to various types of sutures to cause tissue reactions. The results showed that bacterial adherence to braided silk sutures was five- to eightfolds higher as compared to nylon to which the least numbers of bacteria adhered [23]. In another study [9], colonization on various intraoral suture materials from patients microbial having undergone dentoalveolar surgery was investigated. The results showed a larger numbers of bacteria on silk as compared to polyglecaprone 25 [9]. In an experimental study, Leknes et al. [10] investigated the inflammatory responses in oral tissues sutured with silk and ePTFE by recording the presence or absence of bacterial plaque along the suture track. The results showed that bacterial plaque was present in 10 out of the 11 silk and four out of the 11 ePTFE suture channels [10]. These studies may act as possible explanations to the minimum tissue reactions evoked in nylon and polyglecaprone 25 as compared to braided silk sutures. Thus, the different rates of bacterial adherence to various suture materials support the hypothesis that bacterial adherence to sutures plays a significant role in the induction of tissue reactions. Since sutures are immediately contaminated as soon as they contact the oral cavity, it is recommended that sutures should be opened just before being passed through the gingival tissues in order to minimize complications such as stitch abscesses [4].

It is well known that systemic conditions such as poorly controlled diabetes mellitus and cardiovascular disease are directly associated with oral inflammatory conditions [24–28]. Therefore, it may be hypothesized that the massive inflammatory response induced by such confounding factors may “mask” the tissue reactions provoked by the suture material. Data from the clinical studies [8–10, 12, 15], included in the present review, revealed that all participants were systemically healthy; therefore the influence confounding parameters (such as those mentioned previously) may be overruled. In one experimental study [7], tissue reactions to silk, catgut, and Polyglecaprone 25 were investigated in diabetic rats. The results reported similar activities of silk and catgut in the diabetic and control groups [7]. Could this similarity in tissue reactions between the two suture materials be attributed to diabetes control or to the properties of the suture material, remains unclear. Other confounding parameters that may also contribute to oral mucosal inflammation include smoking and use of tobacco products [29, 30]. Nevertheless, due to the lack of data regarding tobacco habits in these studies, the role of tobacco habits as a confounding factor in suture-induced tissue reactions may be a topic to explore for future clinical studies.

## 5. Conclusion

It is still evident that various suture materials used in oral surgical interventions present varying degrees of tissue reactions depending on several factors including surface properties and bacterial adherence properties. The present study emphasizes on the need for careful suture selection of suturing materials for oral surgical interventions.

## Figures and Tables

**Table 1:** Authors, study design, types of oral surgery, suture material sued, statistical methods, and main results of selected studies.

Authors et al. Year	Study design	Subjects	Type of procedure	Suture material used	Main results/conclusions
Yilmaz et al. 2010 [7]	Experimental	Animal model	Oral mucosal surgery (buccal mucosa)	Silk, catgut, and Polyglecaprone 25	Activities of silk and catgut were similar in both diabetic and control groups. All of the materials were well tolerated; however, polyglecaprone 25 had more positive effects on wound healing compared to others.

Sortino et al. 2008 [8]	Clinical	Human	Oral surgery	Silk and PGA	Silk sutures presented a better tension compared to the PGA sutures. There was no difference in the degree of anaerobic bacteria between the two suture materials.

Banche et al. 2007 [9]	Clinical	Human	Dentoalveolar surgery	Silk (resorbable), nylon, polyester, and polyglecaprone 25	A greater quantity of bacteria was found on nonresorbable sutures than on absorbable sutures. Absorbable silk and polyglecaprone 25 exhibited the smallest number of adherent bacteria.

Leknes et al. 2005 [10]	Clinical	Human	Periodontal surgery	Silk and ePTFE	Silk sutures apparently cause a more extensive inflammatory tissue reaction compared to ePTFE sutures.

Leknes et al. 2005 [11]	Experimental	Animal model	Periodontal surgery	Silk and ePTFE	Silk elicits more severe tissue reactions compared to ePTFE.

Otten et al. 2005 [12]	Clinical	Human	Dentoalveolar surgery	Polyglecaprone 25 and polyester	The colonization rate of *Streptococcus intermedius* on both sutures was similar. Growth of *Prevotella intermedia *was faster on polyester as compared to polyglecaprone 25 suture material.

Vastardis and Yukna 2003 [4]	Clinical	Human	Periodontal surgery	Polyglactin 910	A periodontal tissue reaction to the submerged sutures was concluded to be a possible cause of gingival abscesses in these patients.

Charbit et al. 1999 [13]	Experimental	Animal model	Periodontal surgery	Silk, ePTFE, and PLA/PGA	ePTFE and the PLA/PGA sutures were superior to silk in terms of wound healing and inflammatory tissue reactions.

Selvig et al. 1998 [14]	Experimental	Animal model	Periodontal surgery	Silk and chromic gut	Bacterial invasion of the suture track was a common outcome regardless of the material used, but it was particularly prominent for silk sutures.

Abi Rached et al. 1992 [15]	Clinical	Human	Periodontal surgery	Silk, nylon, polyester and perlon	Polyester and perlon induced shorter and less intense tissue reactions compared to silk. Nylon caused the least inflammatory response, with earlier tissue repair.

Okamoto et al. 1990 [16]	Experimental	Animal model	Tooth extraction	Silk, nylon, polyester, and cotton	Nylon showed the best biological results for healing of the dental socket and mucosa. Socket healing was delayed in models with silk and cotton sutures. Healing was only mildly delayed by polyester suture.

Castelli et al. 1978 [17]	Experimental	Animal model	Periodontal surgery	Silk, nylon and cotton	The cheek mucosa reacted to the implantation of cotton and silk with aseptic inflammatory exudates. Nylon did not show an inflammatory response.

Lilly et al. 1972 [18]	Experimental	Animal model	Oral mucosal surgery (buccal mucosa and tongue)	Silk, nylon, PGA, and chromic	The most intense tissue reactions were associated with braided silk sutures. Nylon, chromic, and PGA caused the least tissue inflammatory response.

Lilly et al. 1969 [5]	Experimental	Animal model	Oral mucosal surgery (cheek mucosa and tongue)	Silk, nylon, surgical gut, chromic gut, steel, polyester, and linen	Severe tissue reactions were induced by silk and polyester sutures whereas minimal or no tissue reaction was observed with nylon and polypropylene suture materials.

Lilly 1968 [19]	Experimental	Animal model	Oral mucosal surgery (cheek mucosa and tongue)	Silk, nylon, surgical gut, chromic gut, steel, polyester, and linen	The most intense tissue reactions were induced by silk, cotton, and linen sutures whereas nylon, surgical gut, steel, and chromic showed a mild tissue response.

Lilly et al. 1968 [3]	Experimental	Animal model	Oral mucosal surgery (cheek mucosa and tongue)	Silk, nylon, surgical gut, chromic gut, steel, polyester, cotton, dermal, and linen	The multifilament suture materials (silk, cotton, polyester, and linen) resulted in a significantly higher tissue reaction as compared to the monofilament (steel, nylon, surgical gut, and chromic gut) suture materials.

ePTFE**:** expanded polytetrafluoroethylene; PGA**:** polyglycolic acid suture; PLA**: **polylactic acid suture.

## References

[1] Okamoto T, Rosini KS, Miyahara GI, Gabrielli MF (1994). Healing process of the gingival mucosa and dental alveolus following tooth extraction and suture with polyglycolic acid and polyglactin 910 threads. Comparative histomorphologic study in rats. *Brazilian dental journal*.

[2] Silverstein LH, Kurtzman GM (2005). A review of dental suturing for optimal soft-tissue management. *Compendium of continuing education in dentistry*.

[3] Lilly GE, Armstrong JH, Salem JE, Cutcher JL (1968). Reaction of oral tissues to suture materials. Part II. *Oral Surgery, Oral Medicine, Oral Pathology*.

[4] Vastardis S, Yukna RA (2003). Gingival/soft tissue abscess following subepithelial connective tissue graft for root coverage: report of three cases. *Journal of Periodontology*.

[5] Lilly GE, Salem JE, Armstrong JH, Cutcher JL (1969). Reaction of oral tissues to suture materials. Part III. *Oral Surgery, Oral Medicine, Oral Pathology*.

[6] Wallace WR, Maxwell GR, Cavalaris CJ (1970). Comparison of polyglycolic acid suture to black silk, chromic, and plain catgut in human oral tissues. *Journal of oral surgery*.

[7] Yilmaz N, Inal S, Muğlali M, Güvenç T, Baş B (2010). Effects of polyglecaprone 25, silk and catgut suture materials on oral mucosa wound healing in diabetic rats: an evaluation of nitric oxide dynamics. *Medicina Oral, Patologia Oral y Cirugia Bucal*.

[8] Sortino F, Lombardo C, Sciacca A (2008). Silk and polyglycolic acid in oral surgery: a comparative study. *Oral Surgery, Oral Medicine, Oral Pathology, Oral Radiology and Endodontology*.

[9] Banche G, Roana J, Mandras N (2007). Microbial adherence on various intraoral suture materials in patients undergoing dental surgery. *Journal of Oral and Maxillofacial Surgery*.

[10] Leknes KN, Røynstrand IT, Selvig KA (2005). Human gingival tissue reactions to silk and expanded polytetrafluoroethylene sutures. *Journal of Periodontology*.

[11] Leknes KN, Selvig KA, Bøe OE, Wikesjö UME (2005). Tissue reactions to sutures in the presence and absence of anti-infective therapy. *Journal of Clinical Periodontology*.

[12] Otten JE, Wiedmann-Al-Ahmad M, Jahnke H, Pelz K (2005). Bacterial colonization on different suture materials—a potential risk for intraoral dentoalveolar surgery. *Journal of Biomedical Materials Research B*.

[13] Charbit Y, Hitzig C, Bolla M, Bitton C, Bertrand MF (1999). Comparative study of physical properties of three suture materials: silk, e-PTFE (Gore-Tex), and PLA/PGA (Vicryl). *Biomedical Instrumentation and Technology*.

[14] Selvig KA, Biagiotti GR, Leknes KN, Wikesjö UME (1998). Oral tissue reactions to suture materials. *International Journal of Periodontics and Restorative Dentistry*.

[15] Abi Rached RS, de Toledo BE, Okamoto T (1992). Reaction of the human gingival tissue to different suture materials used in periodontal surgery. *Brazilian Dental Journal*.

[16] Okamoto T, Gabrielli MF, Gabrielli MA (1990). Influence of different types of non-resorbable suture material on the healing of extraction wounds—a histological study in rats. *The Journal of Nihon University School of Dentistry*.

[17] Castelli WA, Nasjleti CF, Diaz-Perez R, Caffesse RG (1978). Cheek mucosa response to silk, cotton, and nylon suture materials. *Oral Surgery Oral Medicine and Oral Pathology*.

[18] Lilly GE, Cutcher JL, Jones JC, Armstrong JH (1972). Reaction of oral tissues to suture materials. IV. *Oral Surgery, Oral Medicine, Oral Pathology*.

[19] Lilly GE (1968). Reaction of oral tissues to suture materials. *Oral Surgery, Oral Medicine, Oral Pathology*.

[20] Reul GJ (1977). Use of vicryl (polyglactin 910) sutures in general surgical and cardiothoracic procedures. *American Journal of Surgery*.

[21] Macht SD, Krizek TJ (1978). Sutures and suturing: current concepts. *Journal of Oral Surgery*.

[22] Ananthakrishnan N, Rao RS, Shivam S (1992). Bacterial adherence to cotton and silk sutures. *The National medical journal of India*.

[23] Katz S, Izhar M, Mirelman D (1981). Bacterial adherence to surgical sutures. A possible factor in suture induced infection. *Annals of Surgery*.

[24] Javed F, Romanos GE (2009). Impact of diabetes mellitus and glycemic control on the osseointegration of dental implants: a systematic literature review. *Journal of Periodontology*.

[25] Bandyopadhyay D, Marlow NM, Fernandes JK, Leite RS (2010). Periodontal disease progression and glycaemic control among Gullah African Americans with type-2 diabetes. *Journal of Clinical Periodontology*.

[26] Javed F, Näsström K, Benchimol D, Altamash M, Klinge B, Engström PE (2007). Comparison of periodontal and socioeconomic status between subjects with type 2 diabetes mellitus and non-diabetic controls. *Journal of Periodontology*.

[27] Inaba H, Amano A (2010). Roles of oral bacteria in cardiovascular diseases—from molecular mechanisms to clinical cases: implication of periodontal diseases in development of systemic diseases. *Journal of Pharmacological Sciences*.

[28] Javed F, Klingspor L, Sundin U, Altamash M, Klinge B, Engström PE (2009). Periodontal conditions, oral Candida albicans and salivary proteins in type 2 diabetic subjects with emphasis on gender. *BMC Oral Health*.

[29] Javed F, Chotai M, Mehmood A, Almas K (2010). Oral mucosal disorders associated with habitual gutka usage: a review. *Oral Surgery, Oral Medicine, Oral Pathology, Oral Radiology and Endodontology*.

[30] Javed F, Altamash M, Klinge B, Engström PE (2008). Periodontal conditions and oral symptoms in gutka-chewers with and without type 2 diabetes. *Acta Odontologica Scandinavica*.

